# Multicrystalline informatics: a methodology to advance materials science by unraveling complex phenomena

**DOI:** 10.1080/14686996.2024.2396272

**Published:** 2024-09-18

**Authors:** Noritaka Usami, Kentaro Kutsukake, Takuto Kojima, Hiroaki Kudo, Tatsuya Yokoi, Yutaka Ohno

**Affiliations:** a Graduate School of Engineering, Nagoya University, Nagoya, Japan; b Institute of Materials and Systems for Sustainability, Nagoya University, Nagoya, Japan; c Institutes of Innovation for Future Society, Nagoya University, Nagoya, Japan; d Graduate School of Informatics, Nagoya University, Nagoya, Japan; e Institute for Materials Research, Tohoku University, Sendai, Japan

**Keywords:** Multicrystalline informatics, grain boundary, dislocation, silicon, machine learning, artificial neural network interatomic potential

## Abstract

Multicrystalline materials play a crucial role in our society. However, their microstructure is complicated, and there is no universal approach to achieving high performance. Therefore, a methodology is necessary to answer the fundamental question of how we should design and create microstructures. ‘Multicrystalline informatics’ is an innovative approach that combines experimental, theoretical, computational, and data sciences. This approach helps us understand complex phenomena in multicrystalline materials and improve their performance. The paper covers various original research bases of multicrystalline informatics, such as the three-dimensional visualization of crystal defects in multicrystalline materials, the machine learning model for predicting crystal orientation distribution, network analysis of multicrystalline structures, computational methods using artificial neural network interatomic potentials, and so on. The integration of these research bases proves to be useful in understanding unexplained phenomena in complex multicrystalline materials. The paper also presents examples of efficient optimization of the growth process of high-quality materials with the aid of informatics, as well as prospects for extending the methodology to other materials.

## IMPACT STATEMENT

We introduce our attempt to explore a methodology for high-performance multicrystalline materials development, multicrystalline informatics, using silicon as a model material.

## GRAPHICAL ABSTRACT

**Figure:**
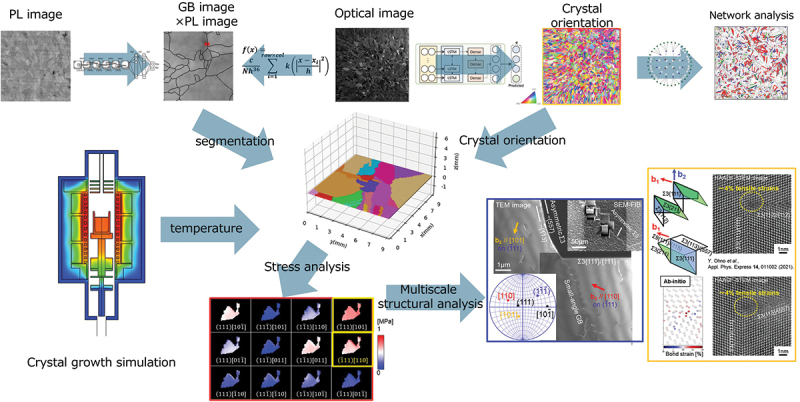

## 1. Introduction

Many practical materials are multicrystalline, and our society is made convenient and comfortable by the continuous development of multicrystalline materials with excellent functions. Multicrystalline materials are characterized by a very complex microstructure consisting of many crystal grains of various orientations and sizes and grain boundaries, which are boundaries between adjacent crystal grains. Because the orientation of crystal grains is often randomly distributed, grain boundaries have diverse atomic arrangements and electronic states depending on their relative orientation relationships. As a result, the properties and functions of grain boundaries are also diverse. A multicrystalline material with a complex distribution of grain boundaries with such diverse properties can be considered an extremely complex system. It is easy to imagine that the macroscopic material properties are highly dependent on the microstructures. The microstructure of a multicrystalline material is not determined solely by its constituent materials but is highly dependent on the process by which the material is fabricated. Scientists have attempted to produce materials with excellent macroscopic properties by controlling the grain boundary characters and bulk nanostructures through the fabrication process in various materials systems [1–8]. However, the question of how to design the microstructures and what process to use to fabricate them still remains unanswered. The complexity of the multicrystalline structure and the diversity of grain boundaries make it difficult to systematize. Thus, we have not yet developed a universal scientific principle to answer the fundamental question, ‘How should we design the microstructures and what process should we use to fabricate it?’

To find a solution to the fundamental question, we proposed a research project titled ‘Multicrystalline informatics toward establishment of general grain boundary physics and realization of high-quality silicon ingot with ideal microstructures’ in 2017. The proposal was accepted, and we began our research project in October 2017. At that time, materials development using data science was already very popular. Most of it, however, focused on discovering new materials with superior properties by means of large numbers of quantum chemical calculations of hypothetical materials containing multiple elements and machine learning models of their properties [9–13]. Our research goals were different. We focused on materials composed of a single element, silicon, with a relatively simple crystal structure. We limited the complex targets requiring data science, such as processing, analysis, and prediction of huge amounts of data, to microstructures and grain boundaries derived from multicrystalline materials. Our belief is that if we can establish a solid research foundation and scientific principles using multicrystalline silicon as a model material, we can expand our research to other complex materials and other crystal systems, and revolutionize materials development methods.

In recent years, cutting-edge research on multicrystalline materials links advanced materials processes, measurement techniques, theoretical calculations, simulations based on physical models, mathematical science, and data science. For instance, scientists have used high-speed calculations to study microstructure formation processes with the help of surrogate models [14–18], generated digital microstructures in virtual space [19–21], and developed machine learning models to predict material properties from digital models [22–24]. These studies have advanced the science of complex and diverse multicrystalline materials at multiple scales. It should be noted that our research project has many unique features that set it apart from these studies.

This paper presents a review of the research foundations established by the project, the evolution of materials science through their integration, and the approach towards creating high-performance materials. The project involved collecting a large number of photoluminescence (PL) images and multi-dimensional optical images of multicrystalline silicon wafers for solar cells. Using image processing and machine learning techniques, a three-dimensional (3D) visualization of the microstructures and dislocation cluster distribution in multicrystalline ingots was developed. Furthermore, crystal orientation distribution was predicted from optical images, and network analysis was used to describe the relationship of microstructures. A machine learning model was also developed to quantify carrier recombination velocities at grain boundaries.

Moreover, a method was established for predicting atomic structures at grain boundaries using an artificial neural network interatomic potential. A method for multiscale structural analysis of feature regions extracted by macroscopic experiments was also developed. The integration of these research bases enabled the creation of realistic 3D multicrystalline models based on data collected from real materials. Furthermore, by linking advanced measurements such as crystal growth simulations, stress analysis, electron microscopy observations, and theoretical calculations of grain boundary atomic structures, phenomena that have been difficult to approach, such as the mechanism of dislocation generation in complex multicrystalline materials, could be elucidated.

For the creation of high-performance materials, a method was developed to efficiently design a growth process that can reduce dislocation density. This was achieved by linking temperature measurement during the material process, solid-liquid interface shape estimation through evaluation of grown ingots, thermo-fluid analysis of the ingot growth process, machine learning models to predict dislocation and stress distribution, and multi-objective optimization using a genetic algorithm. The paper also describes the development of various kinds of research applying the research basis to various materials.

## 2. Research bases of multicrystalline informatics

This chapter introduces various advanced research bases developed by each research group in the first half of the project.

### 2.1. 3D visualization of microstructure and dislocation cluster distribution in multicrystalline ingots

When a semiconductor is exposed to excitation light with higher energy than the band gap, electrons in the valence band get excited to the conduction band, generating holes in the valence band and electrons in the conduction band. These electrons and holes can recombine by emitting light, which is PL, corresponding to the energy difference. If there are crystal defects in the semiconductor, a non-radiative recombination center is formed, which increases the probability of electrons and holes recombining without emitting light, resulting in a decrease in PL intensity. Hence, the spatial distribution of PL intensity can provide information on the spatial distribution of crystal defects in semiconductors [25]. A two-dimensional PL image can be obtained by uniformly irradiating the area of the semiconductor sample with excitation light and capturing the spatial distribution of PL with a camera. This method is widely used in research and development and production control as a non-destructive inspection method for semiconductors as it provides valuable information on crystal quality in a short time without any special treatment or processing of the sample.

In this section, we introduce an example of visualizing the 3D distribution of dislocation clusters in a multicrystalline silicon ingot using PL images [26]. The objects of evaluation are multicrystalline silicon wafers from Kyocera, Japan, which are sliced from a brick that is 156 mm square at the bottom. The brick was cut from a multicrystalline silicon ingot produced by directional solidification of silicon melt. The wafer has a thickness of approximately 180 μm and around 1,000 wafers are obtained from a single brick. The wafer surface shows a metallic luster with periodic linear cutting marks, and it is difficult to recognize individual crystal grains. The entire surface of the wafer was irradiated with 940 nm excitation light and PL images were captured by an InGaAs camera with high sensitivity in the near-infrared wavelength range corresponding to the interband transition emission of silicon (EPL-100s, Hamamatsu Photonics, Japan). The wavelength of the excitation light was set to the indirect transition region with a small absorption coefficient near the absorption edge of silicon to obtain information on the bulk. The wavelength of the light incident on the camera was adjusted using an optical filter, and a portion of the excitation light reflected from the surface was superimposed on the PL image. This was intended to simultaneously obtain information on microstructures in addition to information on crystal defects by enhancing slight differences in reflectance depending on crystal orientation through image processing.

The raw data of PL images collected from multicrystalline silicon wafers contain effects such as unidirectional slice marks on the wafer surface, interference fringes due to scattering of laser light, and emission intensity changes caused by differences in crystal quality and impurity concentration depending on the ingot height. Fourier transform and inverse Fourier transform were used to reduce slice marks. In addition, color density was normalized, and smoothing and unsharp mask processing were applied. Further thresholding was used to extract regions of low PL intensity, and labeling was based on the contiguity of adjacent images to track individual regions. Factors that cause a localized decrease in PL intensity include impurity segregation, electrically active grain boundaries, point defects, dislocations, and so on. However, the low PL intensity regions extracted in this experiment can be determined with high confidence to be dislocation clusters based on their size (several mm in-plane) and their correspondence with the observation results of samples that were etched to detect dislocations.

**Figure 1.**
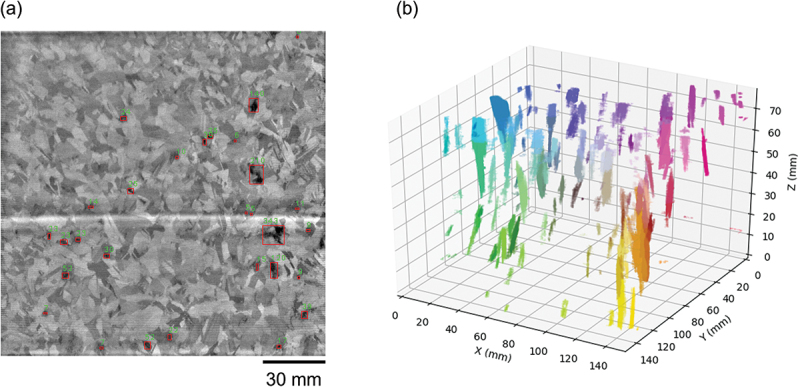
(a) Typical PL image of a 156 mm square multicrystalline silicon wafter after labeling process. (b) distribution of dislocation clusters in a multicrystalline silicon ingot with a base of 156 mm square, which was obtained by image processing of 2D PL images.

Figure 1(a) shows a typical example of a PL image after labeling. Dislocation clusters are surrounded by rectangles. The number above the rectangle indicates the number of pixels in the subthreshold area, which corresponds to the area of the dislocation cluster. 3D visualization of the dislocation clusters was performed by stacking and reconstructing the 2D images after processing as shown in Figure 1(b). To clarify the shape of the dislocation clusters, each cluster is colored. It is seen that a large number of dislocations can be seen in different shapes, sizes, and orientations, providing valuable insights into the quality of the multicrystalline silicon ingot.

### 2.2. Prediction of crystal orientation distribution from optical images

The orientation distribution of multicrystalline materials plays an important role in controlling material properties. The electron backscatter diffraction (EBSD) method is well-known for measuring orientation distribution [27] and is widely used for the analysis of microstructures because it can determine crystal orientation distribution with spatial resolution close to that of a scanning electron microscope. The crystal grain size of multicrystalline materials is produced in a wide range from nanometers to centimeters, depending on the application. EBSD can cover the analysis of materials with grain sizes ranging from sub-microns to millimeters. For the analysis of smaller crystal grain sizes, a technique using diffraction patterns by transmission electron microscope (TEM) has been developed [28]. In general, electron microscopic analysis is restricted to a vacuum chamber of limited size, and samples with diameters larger than ~4 cm generally need to be cut. In addition, several hours of machine time are required per sample to perform orientation analysis with sufficient spatial resolution.

In the manufacturing process of multicrystalline silicon ingot from the melt, the crystal grains with columnar shape are grown from nucleation at the bottom of the crucible. The grain size can be a scale of several centimeters in the horizontal direction and several tens of centimeters in the vertical direction. To obtain crystal orientation information for such structures, it is necessary to analyze dozens of sliced substrates. Therefore, a method that enables crystal orientation analysis at high throughput is desirable.

**Figure 2.**
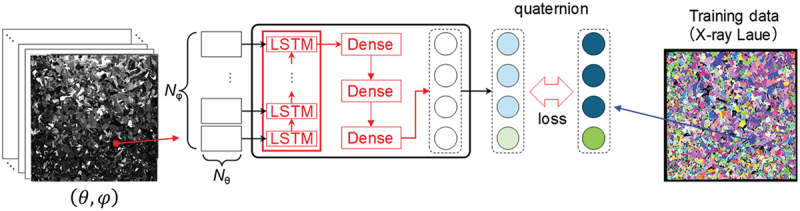
Structure of a machine learning model for predicting crystal orientation from multidimensional optical images.

**Figure 3.**
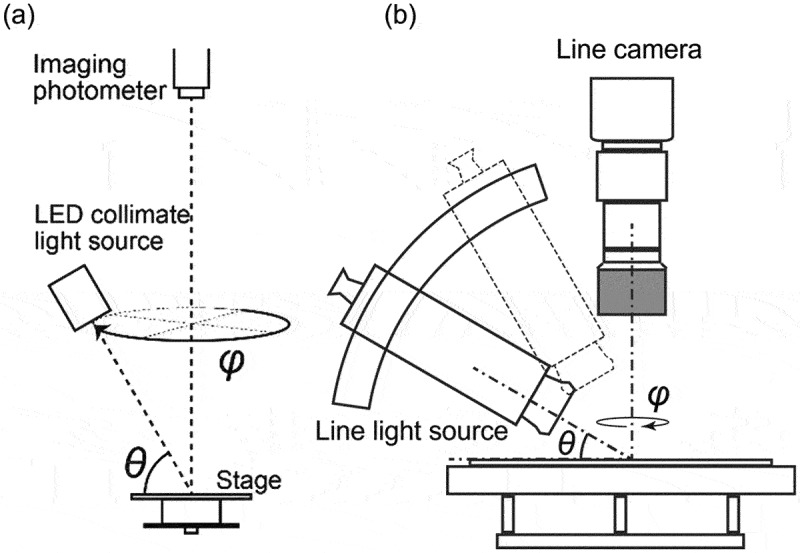
Comparison of two equipment for collecting optical images developed in the project.

In this section, we introduce a method for predicting crystal orientation distribution from multiple optical images using a machine learning model [29]. The main purpose of this section is to solve the crystal orientation estimation problem at a realistic cost using realistic image input by machine learning a neural network model with training data. Based on the knowledge of changes in reflectance in response to changes in light source irradiation, a unique automatic illumination imaging stage was designed as shown in Figure 3(a). To collect data on multicrystalline silicon wafers, a highly parallel white LED light source is installed on a table whose irradiation angle and direction can be controlled, and a high-resolution (6,576 × 4,384 pixels) 2D luminance meter (ProMetric IP-PMY29, KONICA MINOLTA, Japan) is used to collect reflection images of an entire multicrystalline silicon wafer of practical size (156 mm square) while changing the incident light direction. Using the reflectance profiles of each grain obtained from the multidimensional optical images collected by this equipment as input, we constructed an estimation machine learning model for crystal orientation consisting of a recurrent neural network. The output was trained as a quaternion representing the crystal orientation. Quaternions were used because, compared to Euler angles, they were considered more suitable in terms of continuity for learning recurrent neural network maps. For learning, the symmetry of the crystal lattice was considered as a physical constraint. At the beginning of the research and development, data measured at a single elevation angle was used as input, but to improve the prediction accuracy, the dimensionality of the input data was increased by measuring at multiple elevation angles, and the data was expanded by applying a rotation operation to the input vectors and quaternions as shown in Figure 2 [30]. As a result, a median error of 2.4° and an average error of 3.5° were achieved, which is sufficiently accurate for simple orientation prediction.

Although the estimation accuracy was sufficiently high, we proceeded with the design and development of new measurement equipment to further improve the accuracy as shown in Figure 3(b). In the new measurement device, the optical relationship was matched at all measurement points by scanning the sample stage side against a fixed line camera and a line light source set at an elevation angle *θ*. The camera was equipped with telecentric optics, and the light source was set at an elevation angle *θ*. Telecentric optics were used for the camera so that the optical path was restricted to the vertical direction even in the line camera array direction. Since the width of the image is limited to about 4 cm by the telecentric optics, the actuator of the sample stage is driven by three axes (*X-Y-φ*) so that an optical image of any light source azimuth angle *φ* can be captured over the entire sample area. It takes about 30 minutes to take images of a 156 mm square substrate at 5° azimuth angles (from a total of 72 azimuths), and to obtain the reflection characteristics of each grain from 3 × 72 = 216 channels of images with θ ∈ {60°, 45°, 30°} and φ = {0°, 5°,…,355°}. The training of the estimation model has already demonstrated that it exceeds the average accuracy of the conventional method. Since this method can predict crystal orientations simply by collecting optical images in the air without using X-rays or electron beams, it is expected to be applied to various multicrystalline materials.

### 2.3. Network analysis describing generation relationships of multicrystalline structures

In this section, we describe an approach to represent the multicrystalline structure as a network graph by utilizing crystal orientation. As an example, we discuss a twin formation network that predicts the grain generation relationship by generating a network based on the crystal orientation relationship of twins, which are universally present in multicrystalline materials. The handling of the network is formulated by graph theory, a branch of mathematics, and various algorithms can be utilized by implementing them based on the theory.

Twinning introduces mirror-symmetric grains during crystal growth, resulting in the formation of a variety of grain boundaries. The crystal orientation of regions generated by sequential twinning contains information on dynamic changes in the crystal structure. We used the EBSD method (7100-L EBSD, JEOL, Japan) to acquire crystal orientation information of a sample and referred it to the rotational quaternion to analyze the relationship of multicrystalline structure generation.

We focused on analyzing multicrystalline regions with dislocation clusters resulting from twin collisions in a quasi-mono crystal silicon ingot produced in collaboration with the Fraunhofer Institute for Solar Energy Systems [31,32]. We converted the crystallographic orientation to quaternions and used them to determine grain generation relationships. By indexing the orientation relationship for all grains in the analyzed area, we found that multicrystallization likely proceeded solely by the twin formation mechanism.

We extended this method to describe the formation of crystal grain as a network graph. Each grain is considered as a node, and the edges are between grains attributed to the Σ3^n^ rotation operation. As a result, the multicrystalline structure was attributed to multiple connected subgraphs. Since each subgraph originated from the same nucleus, the grain boundaries that serve as interfaces between subgraphs are considered to be random grain boundaries that originated independently of twin formation. This makes it possible to distinguish between random grain boundaries and higher-order Σ3^n^ grain boundaries, which is difficult to do using conventional methods.

Since the generation and propagation of dislocation clusters occur in ingot-scale multicrystalline structures, we attempted to scale up our method to analyze the crystal orientation relationship in 156 mm square wafers. The Lauer scanner was used to measure the crystal orientation of the large-diameter substrates [8]. It is noted that our original method to predict the crystal orientation distribution by the machine learning method described in the last section could also be used.

A challenge of scaling up is that we need to deal with many crystal grains in a large-diameter sample, on the order of hundreds of thousands to millions of pairs. This makes false detection of the twinning relationship when setting a threshold for the returned residuals inevitable. To address this, we formulated a Bayesian inference method to probabilistically evaluate the attribution of the relationship between grains to the twinning relationship.

Using a priori knowledge that the larger the residual δ and the distance *L* between grains, the higher the probability *P* of false detection (*R*), we evaluated the false detection probability distribution *P*(*R*|δ, *L*;Σ ^). Here, Σ ^ denotes the tentatively attributed bicategory relation (Σ ^ ∈ {Σ1,Σ3,Σ9,Σ27a,Σ27b}). Applying Bayes theorem, we obtained

$$P(R|Q;\overset{\wedge }{\Sigma })=\frac{f(Q|R;\overset{\wedge }{\Sigma })P(R;\overset{\wedge }{\Sigma })}{f(Q;\overset{\wedge }{\Sigma })}.$$

**Figure 4.**
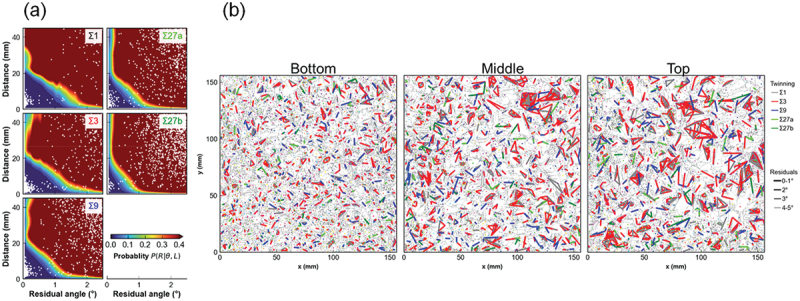
(a) False detection probability distribution *P*(*R*|*θ*, *L*; Σ ^). White dots indicate intergrain relationships. (b) Twinning network due to intergrain relations with *P*(*R*|*θ*, *L*; Σ ^) < 10%. Results on substrates cut from the bottom, center, and top in the ingot in the crystal growth direction from left to right.

Since there are many inter-grain relationships and only a small fraction of them are twinned, *P*(*R*;Σ ^) ≈ 1. We can obtain *f*(δ, *L*| *R*;Σ ^), the probability distribution of δ and *L* for random data, using Monte Carlo method. *f*(*θ*, *L*; Σ ^) was obtained from experimental data, so we could evaluate the false detection probability distribution *P*(*R*|*θ*, *L*;Σ ^) on a substrate cut from the bottom of a multicrystalline silicon ingot as shown in Figure 4(a). The blue region is where the false detection rate is small.

The networks of inter-grain relationships for *P*(*R*|*θ*, *L*; Σ ^) < 10% is shown in Figure 4(b) for substrates cut from the same ingot. Our method visualizes the development of twin networks in macroscopic multicrystalline structures as a result of crystal growth.

### 2.4. Feature extraction of generation points of dislocation clusters

To better understand the mechanism of dislocation cluster generation, it is crucial to identify the crystallographic features of the dislocation cluster generation points. In this section, we introduce our research to classify whether an input image contains dislocation generation points or not by using a transfer learning of a pre-trained convolutional neural network [33]. We also generated PL images from optical and grain boundary images to capture the features of microstructures that contribute to dislocation cluster generation from an informatics perspective. PL images were collected from multicrystalline silicon wafers from the same ingot, and the presence of dislocation clusters was identified by 3D visualization [26]. These images were used as a positive example that includes the point of dislocation cluster generation. Essentially, we attempted to predict the point of dislocation cluster generation by learning the features of the images before being visualized as dislocation clusters in the PL images.

**Figure 5.**
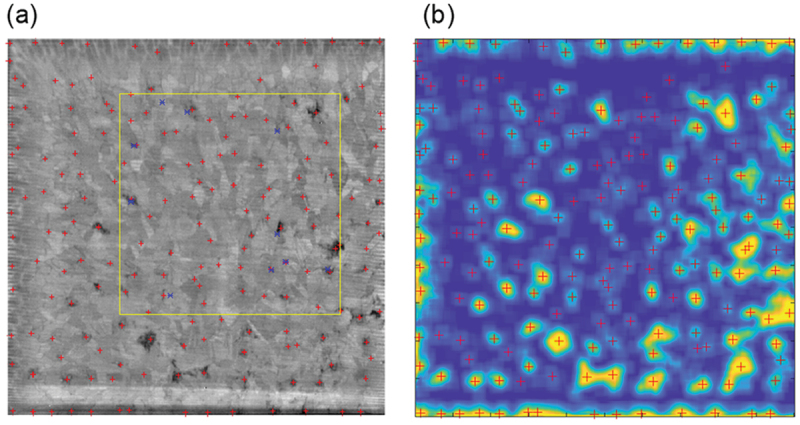
(a) Example of predicted dislocation cluster generation points (peak location: red +, generation points: blue *) (b) heat map display of predicted distribution (high (red) – low (blue)).

To judge the correctness of the prediction, we input a PL image to the learned model to predict whether the input image contains a dislocation generation point and verify whether the upper PL image actually includes dislocation clusters. A PL image of a multicrystalline silicon wafer with a size of 156 mm square was divided into 31 × 31 (approximately 5 mm square, 16 × 16 pixels) input images, and the prediction results for each image were evaluated. To get a pixel-by-pixel visualization of the predicted distribution of dislocation cluster generation points for the entire wafer, the prediction was repeated by shifting the input image by one pixel until the entire wafer was covered. Smoothing peak detection was performed on the obtained distribution, and each peak position was used as the predicted position of the dislocation cluster generation point. The results are shown in Figure 5.

Furthermore, we attempted to generate PL images containing information on dislocation clusters from optical images reflecting microstructures by means of a generative adversarial network. The optical and PL images show the physical processes of microstructure formation and dislocation cluster generation due to the crystal growth process. By using as input a pseudo-color image of three optical images with different illumination directions for the wafer, corresponding to the R, G, and B color channels, the network was trained to reflect the information of the crystal orientation and to transform the optical image to a PL image.

As illustrated by these examples, a machine learning model can estimate the presence of dislocation clusters from the microstructure of a multicrystalline wafer. A model composed of a two-dimensional convolutional neural network had a prediction accuracy of about 89%. However, the internal operations of the neural network are in a black box state, and the basis for the estimation is unclear. To clarify the basis of the model, we analyzed the model using Grad-CAM [34]. Specifically, we computed the gradient that flows into the final convolution layer in the learned model, which represents the magnitude of change that occurs in the output score when a small change is applied to a certain location in the image. From the results of the gradient calculation, we were able to extract important features and learn their relationships with the teacher data.

### 2.5. Quantification of carrier recombination velocity at grain boundaries

One of the important properties of multicrystalline silicon for solar cells is the electrical properties of grain boundaries, especially the carrier recombination velocity. Since the carrier recombination velocity strongly depends on the grain boundary structure, it is necessary to analyze it on an individual grain boundary and even on a position-by-position basis based on local evaluation. Although PL images on a macroscopic scale are generally used to evaluate the carrier recombination velocity [35], we have attempted to construct a quantitative evaluation method using microscopic PL images in order to assess the carrier recombination velocity with higher spatial resolution.

Specifically, we developed a two-dimensional carrier simulation model that considers the inclination of grain boundaries and developed a method for quantitative evaluation of carrier recombination by fitting the carrier recombination velocity at grain boundaries and carrier diffusion length within grains as fitting parameters to the PL intensity profile across grain boundaries obtained by experiment [36]. However, repeated simulations were required for fitting, and the computational time required for analysis was a bottleneck in the evaluation.

**Figure 6.**
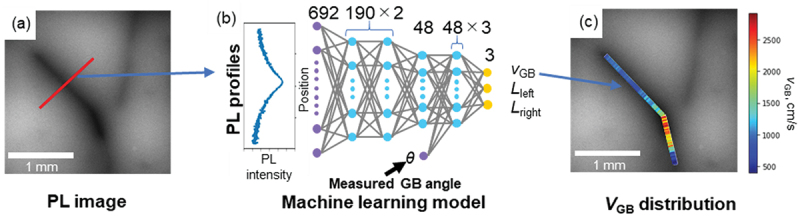
(a) Example of PL image, (b) machine learning model, and (c) spatial distribution of predicted carrier recombination velocity.

The simulation model above mentioned was used to prepare a data set of physical properties (carrier recombination velocity, carrier diffusion length, and grain boundary tilt angle) and PL intensity profiles. The data set was then used for machine learning to create a fast prediction model [37]. First, 3,000 PL intensity profile simulations were performed with random values of physical properties, and the data was split into a 60% training set, 20% validation set, and 20% test set. A feed-forward neural network was used for machine learning, with the experimentally observable PL intensity profile and grain boundary tilt angle as inputs, and the carrier recombination velocity and carrier diffusion length as outputs (Figure 6(b)). The inputs and outputs of this machine learning model are the reverse of those of the simulation, *i.e*., the carrier recombination velocity is obtained directly from the PL intensity profile obtained by the measurement, *i.e*., the inverse problem is solved directly. The prediction accuracy of the machine learning model, obtained after adjusting the network structure and learning parameters, is 0.245 in powers of 10 of the carrier recombination velocity (cm/s). This level of accuracy is sufficient for the purpose of grain boundary evaluation. The analysis time was significantly reduced from 198 s to 0.02 s using the conventional method based on simulation fitting. The significant reduction in analysis time by machine learning allows for the continuous evaluation of a large number of PL intensity profiles along grain boundaries. This allowed us to quantitatively determine the local increase in carrier recombination velocity in the grain boundary fold region (Figure 6(c)). This also allowed for a detailed investigation of the relationship between the grain boundary structure and the electrical properties of the grain boundary.

The structure of the machine learning application conducted consists of creating a machine learning model, which is an inverse problem of the simulation, and applying experimental data to it. This process is common in material evaluations, not limited to PL image analysis. Therefore, we consider that this is a good example of machine learning application for material evaluation.

### 2.6. Prediction of atomic structure using artificial neural network interatomic potentials

**Figure 7.**
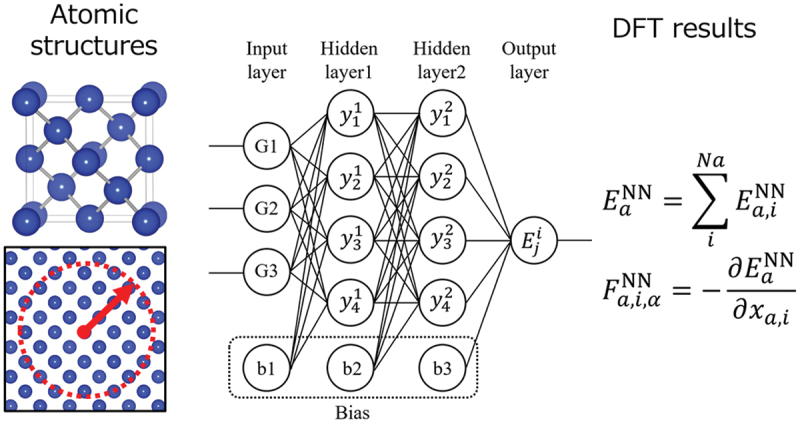
A schematic of ANN potential.

Figure 7 shows a schematic of the artificial neural network interatomic potential (ANN potential) implemented in this study [38], which is a nonlinear function with many tuning parameters and is a machine learning model with excellent function approximation capabilities. Based on Behler’s previous work [39], we implemented a feed-forward network with two hidden layers each having 24 nodes. Although the input data is a crystal structure, it is converted to input values using structural descriptors that encode the atomic environment of each atom. The input values were obtained using the two- and three-body symmetry functions devised by Behler. Supervised learning was then performed by mapping the output of the ANN and the first-order derivative of the output with respect to the atomic positions to the energy of the system and the force on the atoms, respectively, obtained from the DFT calculation. Batch learning using the Levenberg-Marquardt method was used as the learning algorithm. DFT calculations (VASP code [40–42]) based on the projector augmented wave (PAW) method [43,44] were performed to generate training and test datasets. The wave functions were expanded in the plane wave basis set with an energy cutoff of 500 eV. A modified version of the generalized gradient approximation (GGA-PBEsol) formulated by Perdew, Burke, and Ernzerhof [45] was used to calculate the exchange correlation energy. The convergence condition for the total energy in the self-consistent field method was set to 10^−6^ eV, and the number of meshes in the *k*-point sampling was 6 × 6 × 6 for a cubic unit cell for the diamond structure. DFT-MD calculations based on Parrinello-Rahman dynamics were performed during DFT data collection [46,47]. This calculation was performed under NVT and NPT conditions with a time step of 2 fs.

In order to generate training data containing a variety of atomic environments, point defects and surfaces as well as perfect crystals were considered. We also considered symmetric tilt grain boundaries with the $\left[001\right]$ and $\left[1\overset{ˉ}{1}0\right]$rotation axes. Using these cells as references, we randomly displaced atoms and changed lattice constants, and performed about 10–20 steps of structural relaxation using these cells as initial structures. Snapshots were then added to the training data. DFT-MD calculations were also performed at 600, 800, 1000, and 1200 K, and these snapshots were also added to the training data. Finally 11,770 data for the energy of the system and 3,210,000 data for the force on the atoms were used as the training data. In order to determine whether the ANN potential is applicable to general grain boundaries, we tested whether the prediction ability holds for grain boundaries that are not included in the training data. The test data was generated in the same way as for the training data described above. The errors for these grain boundaries were then evaluated.

The mean absolute error (MAE) for all the training data was 5.7 meV/atom for energy and 111 meV/Å for force on atoms. These values are expected to be sufficient for performing ordinary molecular simulations with reasonable accuracy. The trained ANN potential was then used to evaluate the errors for the grain boundaries of the test data. Although an exact comparison is difficult due to the variation in the length of the bulk region for each grain boundary model, almost all data are well predicted except for the Σ11(113)/$\left[1\overset{ˉ}{1}0\right]$ grain boundary. The MAE of the energy is 1.7–3.1 meV/atom, which is close to the MAE of the training data. Therefore, it can be said that the ANN potential maintains the excellent predictive ability even for grain boundaries absent in the training data. On the other hand, for the Σ11(113)/$\left[1\overset{ˉ}{1}0\right]$ grain boundary, the ANN potential underestimates the value of the DFT calculation overall. The MAE was also 11.0 meV/atom, which is about twice the MAE of the training data. This is thought to be due to the fact that only five types of symmetric tilt grain boundaries were added to the training data, and therefore the atomic environment of the grain boundaries was not fully covered. This point can be resolved by further expanding the training data and searching for atomic environments that are not sufficiently interpolated using the ANN potential that has been trained once.

It was shown that the ANN potential after training retains the predictive ability for the grain boundaries of the test data. However, this kind of error evaluation corresponds to a single point calculation with the ANN potential for precalculated DFT calculation data. On the other hand, when performing molecular simulations with the ANN potential, it is necessary to first evaluate the energies and forces on atoms along the energy surface of the ANN potential in the absence of DFT calculation data. Therefore, the calculations must be stable with sufficiently small errors in all atomic environments of interest. To verify this point, it is necessary to perform molecular simulations with the ANN potential and evaluate the errors with DFT single-point calculations using the resulting atomic structures.

**Figure 8.**
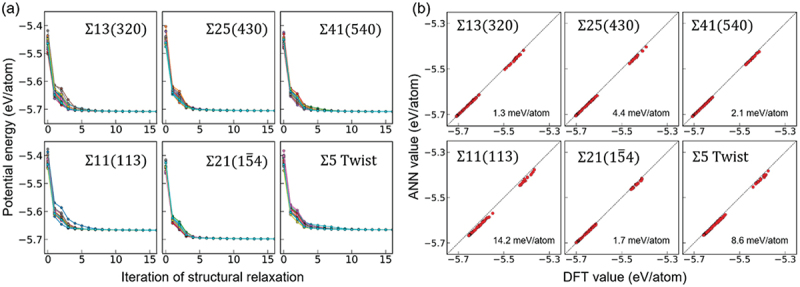
(a) Results of structural relaxation with ANN potentials by randomly displacing atoms based on the grain boundaries of the test data. (b) Results of evaluating the error between the ANN potential and the DFT calculation using the structure in the process of structural relaxation.

With the above point in mind, the ANN potential was used to perform structural relaxation calculations on grain boundaries for the test dataset. The calculation cells were created by randomly displacing the atomic positions with reference to the respective grain boundary structures obtained in advance by DFT calculations. Several cells were fabricated for one grain boundary in the same manner, and they were structurally relaxed by the ANN potential, as shown in Figure 8(a). The results show that the energy for any of the grain boundaries approached convergence in a few tens of steps, and no divergence of the calculations was observed. Figure 8(b) shows the results of a single point DFT calculation of a snapshot of the structural relaxation and the evaluation of the error. From this figure, it can be seen that the result of structural relaxation by the ANN potential is comparable to the error of the training data, indicating that sufficient accuracy is maintained. Therefore, it is expected that the calculation time can be significantly reduced by narrowing down the candidate grain boundary structures in advance using the ANN potential and finally verifying the results using the DFT calculation. On the other hand, the MAE for the Σ11(113)/$\left[1\overset{ˉ}{1}0\right]$ grain boundary was 14.2 meV/atom, which is nearly three times the MAE of the training data. In such a case, a large error in grain boundary structure refinement is expected. Therefore, expansion of the training data is necessary to achieve even higher accuracy.

### 2.7. Multiscale analysis of dislocation sources

As described in Secs. 2.1 and 2.2, we can visualize 3D distribution of dislocation clusters and GBs in multicrystalline ingots of 10^2^ mm scale, by using PL and optical imaging techniques assisted by machine learning. The 3D modeling enables us to determine the locations and characters of the GBs acting as dislocation sources within a spatial resolution of less than sub-millimeters. The wafers with the determined GBs are etched chemically so as to form etch pits of dislocations, and the spatial distribution of dislocations around the GBs, as well as the morphological characteristics of the GBs, are examined by scanning electron microscopy (SEM). We can expect the GB segments acting as dislocation sources within a spatial resolution of less than sub-micrometers. The characters of each GB, including the crystallographic orientation of the grains separated by the GB, can be determined by optical imaging techniques denoted in Sec. 2.2, and many GBs acting as dislocation sources would be tilt boundaries. Thin foils with one of the GBs, about 30 µm × 30 µm × 0.5 µm in size, are cut from a wafer so that the tilt axis of the GB is normal to the foil surfaces, by a micro-sampling technique using a focused ion beam (FIB) system equipped with SEM (Helios NanoLab600i, FEI, Japan). Mesoscopic morphology of the GB and dislocations are examined by conventional transmission electron microscopy (TEM). The GB have a staircase-like structure composed of stable GB facets with low GB energy, and the Burgers vectors of the dislocations emitted from the GB are parallel to the facet junctions, suggesting that the facet junctions would be the origin of dislocation generation. Therefore, atomic arrangement at the facet junctions is examined by high-angle annular dark-field scanning TEM (HAADF-STEM), with a spatial resolution of 0.1 nm (JEM-ARM200F, JEOL. Japan). By using the HAADF-STEM data, supercells with the GB, containing fewer than 500 silicon atoms, are constructed. The atomistic structure around the facet junctions is examined by performing *ab-initio* calculations with the supercells. *Ab initio* calculations revealed tensile bond strains of more than 2% (or bond extension more than 0.006 nm) along the facet junctions, and this results in the reduction of the yield stress for dislocation generation, more than 10% lower than that of perfect Si crystals. As a result, by using a multiscale analysis on a scale ranging from 10^2^ mm to 10^−3^ nm, it is found singularities in the GB segments, i.e. facet structures that would reduce the yield stress for dislocation generation, thus revealing the existence of a dislocation generation mechanism unique to the multicrystalline structure.

## 3. Deepening of materials science and creation of high-performance materials through integration of research bases

In this chapter, we show that the integration of the advanced research bases introduced in Chapter 2 will enable us to unveil phenomena in complex multicrystalline materials and systematize the relationship between grain boundary structure and physical properties, which have been difficult to approach in the past, and contribute to the deepening of materials science and the creation of high-performance materials.

### 3.1. Unveiling generation mechanism of dislocations

We captured optical images that have information about crystal orientation and tried to create a three-dimensional model that combines data on grain boundaries and crystal orientations. To collect the data, we treated multicrystalline wafers with an alkaline solution to create a textured structure on the crystal surface. We used an originally developed luminance measurement equipment to obtain a multidimensional optical image by capturing an image each time the light source position was rotated by a specific angle. We then applied the mean shift method to the luminance value vector at each pixel for grain segmentation. After that, we stacked the results of grain expansion processing and manual correction to get a three-dimensional grain segmentation image. For the crystal orientation information, it is possible to use the prediction results from the machine learning model introduced in 2.2. Elastic constants that take into account the anisotropy of silicon are given to each crystal grain, and stress analysis is performed under conditions that are linked to the temperature distribution of the crystal growth simulation results [48]. Shear stresses along 12 equivalent slip systems were calculated from the analysis results, and the largest one was mapped. The results showed that the shear stresses were concentrated near the grain boundaries where dislocation clusters were generated. The calculated main slip system in the dislocation-cluster-generated grains is in the [110] and [101] directions on the (11̅1) plane, which is consistent with the direction of propagation of dislocation clusters obtained by microscopic PL images and the Burgers vector of dislocations obtained by TEM observation. Furthermore, electron microscopic observation revealed that the grain boundaries bend while forming a mesoscopic staircase-like structure with stable grain boundary segments with low grain boundary energy, from which dislocations originate. Even at the random grain boundary triple point where dislocation clusters were generated, the propagation plane of the random grain boundary was bent to form a facet structure in the region where it contacted the ∑3 grain boundary. These results indicate that the formation of facet structures associated with grain boundary bending and the stress concentration at the facet junctions are one of the universal mechanisms of dislocation generation.

**Figure 9.**
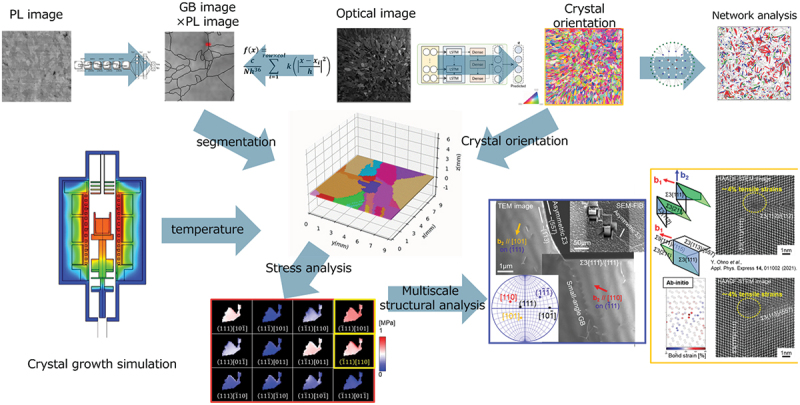
Integration of the advanced research bases developed in the project.

In this study, as shown in Figure 9, data collected from real materials, machine learning, and crystal growth simulations are used in conjunction to conduct the analysis for unveiling phenomena in complex multicrystalline materials, which can be considered an original research method [49].

### 3.2. Relationship between grain boundary structures and carrier recombination velocity

**Figure 10.**
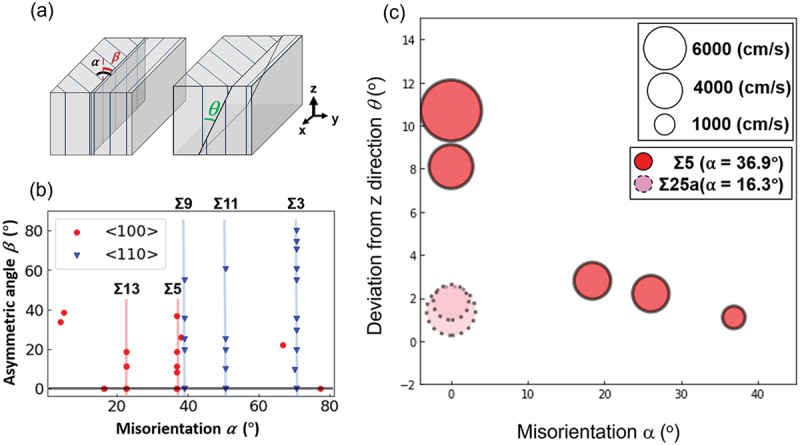
(a) Three parameters to characterize the geometry of a grain boundary (b) prepared artificial grain boundaries (c) estimated carrier recombination velocity at grain boundaries with various α and θ.

In multicrystalline silicon, it is widely known that the grain boundary structure has a strong influence on the electrical properties of grain boundaries. However, most of the previous studies dealt only with relative orientation differences, α in Figure 10(a) [50,51]. In actual grain boundaries, the effects of asymmetry angle (rotation of the grain boundary around the common crystallographic orientation axis, β in Figure 10(a)) and deviation angle (inclination of the grain boundary with respect to the common crystallographic orientation axis, θ in Figure 10(a)) have rarely been examined [52]. The reasons for this are the difficulty in obtaining samples with systematically varied grain boundary structures and the lack of local and quantitative characterization methods. We applied grain boundary structure control using seed crystals to the former issue and high-speed evaluation utilizing machine learning as described in 2.5 to the latter issue, thereby enabling detailed clarification of the effects of grain boundary structures. Figure 10(b) summarizes the structures of 34 types of grain boundaries that we fabricated. The crystal orientation in the ingot growth direction was < 100> or < 110>, and several grain boundaries with different asymmetry angles were fabricated at the major coincident site lattice grain boundaries (Σ grain boundaries) in each orientation. Detailed growth conditions are found elsewhere [53]. It should be noted that the grain boundary structures spontaneously changed during growth depending on the solid-liquid interface geometry [54].

Figure 10(c) summarizes the relationship between grain boundary structure and carrier recombination velocity at 12 mm from the seed crystal for < 100> oriented grain boundaries. It can be seen that the impact of θ on the carrier recombination velocity is larger than that of α. This trend was also confirmed at different positions from the seed crystal. Overall, the trend in the magnitude of effect on the carrier recombination velocity was θ > α > β. This trend may be explained in terms of the local reconstruction of grain boundaries with enhanced local distortion by θ. We have reached this conclusion based on the < 100> grain boundaries evaluated so far. It is necessary to assess a wider range of grain boundaries and conduct microscopic structural evaluations and calculations.

### 3.3. Creation of high-performance materials

We have proposed a high-quality quasi-monocrystalline ingot growth technique that suppresses polycrystallization and dislocation clustering in the product part by introducing functional defects combined with artificial grain boundaries at the seed crystal. We have demonstrated its usefulness with a practical-size ingot, from which four blocks for wafers can be obtained [31]. In addition, we investigated a method that combines a surrogate model of the crystal growth simulation with an optimization algorithm with the aim of further reducing dislocation density by optimizing the growth process parameters [48,55]. Functional defects intentionally introduced into the joints of seed crystals are meant to surround dislocation-prone grain boundaries with grain boundaries that block dislocation propagation [56]. This aims to concentrate dislocation clusters at the cut zone when the ingot is cut into bricks, which reduces stress and ensures high-quality crystals in the final product. It is noted that the design of these defects is based on knowledge obtained from visualizing dislocation clusters in 3D and predicting the probability of dislocation generation described in Chapter 2.

**Figure 11.**
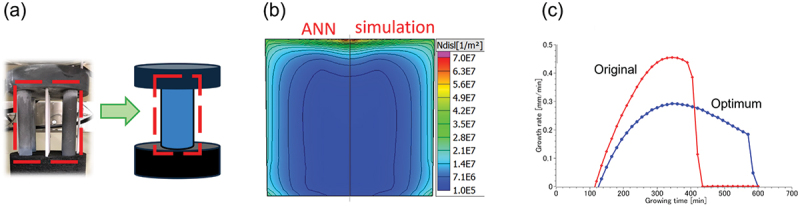
(a) An example of the geometry transformation for simulation (b) comparison of the dislocation density in an ingot predicted by the ANN model and simulation (c) comparison of the growth rate of the silicon grown using original recipe at a constant pulling rate of the crucible and optimum one with time-dependent pulling rate.

The optimization framework consists of four stages: lab-scale growth experiments, crystal growth simulations, the creation of a surrogate model for the simulations using an ANN, and optimization using genetic algorithms. The lab-scale furnace permits growing small ingots of approximately 100 mm square. Three independently controllable heaters are installed to achieve a variety of temperature distributions. In the crystal growth simulation, the entire system is approximated by a two-dimensional axisymmetric model. For example, Figure 11(a) shows a picture of a susceptor of a crucible in comparison with a computational model. In this case, the governing equation for vertical heat conduction is taken into account, and the geometry is transformed to a single cylinder shape so that the cross-sectional area is equal to that of four cylinders. The calculations provide information on the changes in the shape of the solid-liquid interface during the growth process and the spatial distribution of equivalent stress and dislocation density after ingot growth. Crystal growth has been empirically performed unidirectionally by pulling down the crucible at a constant rate while keeping the temperature of each heater constant. On the other hand, simulations were conducted for various combinations of heater temperatures and crucible pull-down speeds, allowing for time variation. The results were then used as data to train the ANN and create a surrogate model for the simulations. Figure 11(b) compares the ANN prediction and simulation for dislocation density. It is seen that ANN can well reproduce the simulation result. To design the optimal process parameters, we used the multi-objective genetic algorithm NSGA-II to obtain the Pareto front with the minimum residual stress, dislocation density, solidification time, and average dislocation density. Since the primary goal of our process design is to reduce dislocation density, we selected the optimal recipe with the lowest dislocation density, relatively short solidification time, and low residual stress. The resultant growth rate for the optimum recipe was compared with the original one in Figure 11(c).

To validate the optimal recipe, simulations were performed. In terms of dislocation density, the optimal recipe resulted in a lower maximum dislocation density of 2.2 × 10^7^ m^−2^, which was reduced by 84% at the top part of the ingot. In the single-crystal region, the dislocation density was also reduced from 5.5 × 10^6^ m^−2^ in the original ingot to 3.8 × 10^6^ m^−2^ in the optimized ingot.

When ingots were actually grown according to the predicted optimal recipe, the initial growth interface was much flatter than in the original case for the optimal recipe. Due to the aspect ratio of the small crucible, the growth interface changed to a concave shape as the growth progressed, but the growth interface became flat at the end of the growth phase. In fact, a decrease in dislocation clusters and suppression of polycrystallization at the top of the ingot were observed in the optimal recipe, and the usefulness of this method was experimentally demonstrated.

## 4. Future prospects and concluding remarks

We reviewed our project on ‘multicrystalline informatics’, which is a scientific principle that offers universal guideline for high-performance multicrystalline materials with complex microstructures and diverse grain boundaries. We have established a research approach using silicon as a model material, and the findings are applicable to other materials as well.

The system for collecting multidimensional reflection images from multicrystalline materials and the orientation prediction model based on machine learning, as introduced in section 2.4, can be used for simple measurement in the air without requiring X-rays or electron beams. These methods are expected to be applicable to various materials and for developing measurement systems. Our next steps involve increasing the resolution of the measurement and creating a high-precision orientation prediction model to expand the range of target materials. We also plan to collaborate with manufacturers to bring the measurement system to market. As an example, we applied this method to analyze the behavior of grain boundary growth in the melt growth of Mg_2_Si. Our findings revealed that the preferential growth orientation changes throughout the growth process. When combined with crystal growth simulations, we clarified that the increase in undercooling with growth significantly affects the change in the microstructure [57].

The network analysis of multicrystalline structures described in section 2.5 was initially developed for silicon, a cubic crystal, and is now being expanded to include other crystal systems such as tetragonal crystal structure. We have observed that the expansion of the twin network is limited when analyzing the orientation data of the iron-based superconductor BaFe_2_As_2_ prepared by the sintering process [58,59]. Furthermore, we have initiated a study to examine the relationship between features of the network graph, which visualizes the orientation fluctuations from the c-axis, and the superconducting current density.

ANN potentials introduced in section 2.6 could be shared with other research institutes for their use. These potentials, along with the weight and bias parameters, the learning program, and the molecular simulation program, can be applied to compound semiconductors [60], metals [61], and oxides [62], where grain boundaries play a crucial role in material properties. We have successfully developed ANN potentials with strong prediction capabilities for grain boundaries that are not included in the training data, allowing for comprehensive prediction of grain boundary structures prior to DFT calculations. This approach also enables detailed atomic structure analysis and electronic structure analysis through DFT calculations based on the obtained grain boundary structures. By extending this methodology to complex grain boundaries, we aim to gain insight into the atomic structure and properties of general grain boundaries across a wide range of materials.

We have integrated theory, computation, and advanced measurement to create realistic 3D polycrystalline models based on data collected from real materials. By further expanding our research methodology to include cutting-edge techniques such as operand measurement and process modeling, we aim to contribute to process informatics research and expand the materials search space to include metastable and multicomponent materials. This space can be infinitely expanded by adding different types and compositions of constituent elements along with process parameters. This hyperspace can be likened to a vast materials search space, similar to the universe, where unexplored materials with innovative functions exist. Throughout history, superior materials have transformed society multiple times. If we can develop a new methodology to discover multicrystalline structures with exceptional functions and their creation process from this vast materials search space, it will be possible to contribute to innovation in various industrial fields.
